# Two-step cytoreductive surgery and hyperthermic intraperitoneal chemotherapy for pseudomyxoma peritonei with high peritoneal carcinomatosis index

**DOI:** 10.1186/s12957-021-02171-z

**Published:** 2021-02-23

**Authors:** Bertrand Trilling, Alexandre Brind’Amour, Raphael Hamad, Jean-Francois Tremblay, Pierre Dubé, Andrew Mitchell, Lucas Sidéris

**Affiliations:** 1 Department of Surgery, Hôpital Maisonneuve-Rosemont, Université de Montréal, 5415 boul. de l’Assomption, Montréal, Québec, H1T 2M4 Canada; 2 Department of Pathology, Hôpital Maisonneuve-Rosemont, Université de Montréal, Montréal, Canada

**Keywords:** Bulky pseudomyxoma, Two stage complete cytoreductive surgery, High peritoneal carcinomatosis index, Peritoneal metastasis resectability

## Abstract

**Background:**

Complete cytoreductive surgery (CRS) combined with hyperthermic intraperitoneal chemotherapy (HIPEC) is the only curative treatment for pseudomyxoma peritonei (PMP) arising from the appendix. High peritoneal carcinomatosis index (PCI) is associated with an increased risk of surgical complications. The objective of this study was to present the results of a planned two-step surgical strategy to decrease postoperative morbidity and improve resectability of patients with very high PCI.

**Methods:**

All consecutive patients who underwent a planned two-step surgical approach for PMP between January 2012 and March 2020 were retrospectively included. This approach was offered for patients with low-grade PMP with PCI > 28 for which feasibility of a complete CRS in one operation was uncertain. The first surgery included a complete CRS of the inframesocolic compartment and omentectomy. HIPEC was delivered at the second surgery, after complete CRS of the supramesocolic compartment. Postoperative morbidity was assessed using the Clavien-Dindo classification and survival results were also collected.

**Results:**

Eight patients underwent the two-step approach. The median PCI was 33 (29–39) and the median time between the two procedures was 111 days (90–212 days). One patient was deemed unresectable at the second surgery. The rate of major morbidity was 0% for the first step and 25% for the second step, with no mortality. Median follow-up was 53.8 months (3–73 months).

**Conclusion:**

A two-step surgical management for low-grade PMP patients with very high PCI is safe and feasible, with acceptable postoperative morbidity and no compromise on oncological outcomes.

## Introduction

Pseudomyxoma peritonei (PMP) is a rare condition characterized by intraperitoneal dissemination of mucinous neoplasia affecting about 1 patient per million population per year [1]. Its main origin is the appendix, but it may also arise from the ovaries or other intraperitoneal organs. Although this disease is usually slowly progressive, its natural course is fatal due to increased abdominal distension, intestinal obstruction by extrinsic compression, or respiratory failure [2].

PMP may present with different histologic subtypes. Ronnett et al. classified appendiceal peritoneal carcinomatosis as diffuse peritoneal adenomucinosis (DPAM), peritoneal mucinous carcinomatosis (PMCA), or having intermediate features (PMCA-I) [3]. A simpler consensus classification scheme has since been proposed by PSOGI (Peritoneal Surface Oncology Group International), which distinguishes low-grade appendiceal mucinous neoplasms (LAMN) from high-grade appendiceal mucinous neoplasms (HAMN) [4].

The introduction of complete cytoreductive surgery (CRS) followed by hyperthermic intraperitoneal chemotherapy (HIPEC) has dramatically improved outcomes for patients with PMP [5]. In a large multicenter retrospective cohort of 2298 PMP patients who underwent CRS and HIPEC, respective 5- and 10-year overall survival (OS) for patients with DPAM were 81% and 70%, compared to 59% and 49% for PMCA and 78% and 63% for those with intermediate features [6]. In a more recent study [7] of 265 patients with PMP who underwent CRS and HIPEC, the 10-year OS was 63% for LAMN and 40% for HAMN.

The balance between surgery-related morbidity and oncological benefit is one of the main concerns of surgical oncologists when it comes to major oncologic procedures. The peritoneal cancer index (PCI) [8] is a quantitative measure that describes the extent of peritoneal disease at surgery. A very high PCI may be considered a contraindication for CRS, as it significantly increases the morbidity and mortality associated with the procedure [9], along with a significant impact on quality of life [10]. While CRS and HIPEC have improved oncological outcomes for patients with PMP, this procedure is associated with high operative mortality (0–18%) and morbidity (33–56%) [11–14]. For PMP patients with unresectable disease, debulking only has been accepted as a palliative procedure, as it improves overall survival and quality of life with acceptable morbidity for symptomatic patients [15].

Due to the more indolent nature of low-grade PMP compared to other peritoneal malignancies, our group has developed a two-step surgical approach for the curative treatment of patients with very high PCI in order to decrease the morbidity and mortality associated with the procedure and to offer a better alternative than debulking alone to patients with unresectable disease. The objective of this study was to assess the safety of this approach and to report its influence on perioperative and oncologic outcomes.

## Methods

### Study design and patient selection

A retrospective study of all patients who were deemed fit for a two-step surgical approach for PMP between January 2012 and March 2020 at an academic tertiary center specialized in peritoneal surface oncology was performed. The study was approved by the ethics committee of the CIUSSS de l’Est-de-l’Île-de-Montréal based on the nature of data collected. All patients were assessed for surgery following the Canadian HIPEC Collaborative Group guidelines [16] and signed informed consent.

The selection of the patients deemed fit for a two-step surgical approach was made on a case-by-case basis, either preoperatively, based on imaging and laparoscopic evaluation, or intra-operatively, if the PCI had been underestimated at preoperative work-up. This approach was considered only for low-grade PMP patients (DPAM, PMCA-I, or LAMN) with very extensive disease, which can be defined as a PCI ≥ 28 [17], in which complete CRS was deemed achievable with two surgeries and for which the disease was presumed to be indolent and therefore, unlikely to flare up between procedures. Patients with histological diagnosis of PMCA, extraperitoneal metastases, or surgery for recurrence were not considered for this approach.

### Two-step surgical management

All patients included in this study were operated on by at least one of the two senior board-certified surgeons (LS, PD). The first step consisted of a complete CRS of the inframesocolic compartment without HIPEC. Colonic resections, greater omentectomy, lower abdomen peritonectomy, and removal of any pelvic or retroperitoneal mass were usually performed at this stage. Any supramesocolic dissection was minimized in order to prevent future adhesions. The abdomen was first approached through a midline xyphopubic incision. After complete exploration of the abdominal cavity, the PCI was scored as previously described [18]. If DPAM, PMCA-I, or LAMN diagnosis was not already confirmed, an intra-operative frozen pathology examination was performed. After a thorough adhesiolysis, the PCI was measured and the small bowel was assessed carefully. A total colectomy with ileo-rectal anastomosis below the level of Douglas’ pouch was performed in most cases. The creation of a diverting loop ileostomy was left at the discretion of the operating surgeon depending on local conditions. During the omentectomy, the gastroepiploic vessels were preserved if no macroscopic disease was found. The gastro-splenic ligament was usually left intact and resected at the second procedure. Patients were then left to recover until the second step was performed a few months later.

The second step consisted of a thorough adhesiolysis of the inframesocolic compartment, with removal of nodules that could have recurred in the meantime. Frozen pathology was performed in case of suspected invasive nodules. The pelvis was dissected to enable adequate intraperitoneal chemotherapy diffusion. Major recurrence and invasive nodules were contraindications for CRS and HIPEC. A complete CRS of the supramesocolic compartment was realized, sometimes including a gastrectomy and a splenectomy, followed by HIPEC with oxaliplatin according to the institution’s protocol [19]. In our institution, the need for a total gastrectomy, along with a total colectomy, is considered reasonable if no small bowel resection is needed.

### Pathology

Grading of both primary and peritoneal deposits was initially performed by an experienced pathologist using Ronnett’s classification [3]. DPAM was characterized by the presence of scant low-grade adenomatous mucinous epithelium within abundant extracellular mucin and associated fibrosis. PMCA displayed the cytologic and architectural features of higher-grade mucinous carcinoma associated with extracellular mucin, often with invasive components. PMCA-I presented with both DPAM and PMCA characteristics. For the purpose of this study, pathology slides were reviewed using PSOGI classification and specimens were further categorized into LAMN or HAMN.

### Follow-up

Patients were seen in the outpatient clinic at 4-month intervals. CT scans were performed every 4 months for the first 2 years, bi-annually for an additional 3 years, and yearly thereafter to assess for remission status or disease progression.

### Outcomes

Data on baseline characteristics, peritoneal pathology, operative details, complications, and long-term follow-up were extracted from chart review. The primary outcome for this study was postoperative complications, as defined by the Clavien-Dindo Classification [20]. Major morbidity was defined as Clavien-Dindo grades III-IV, while minor morbidity included Clavien-Dindo grades I-II. All complications noted in the patients’ charts and directly related to the surgical procedure were included. Prolonged ileus was defined as lack of return of bowel function after 6 days or need for nasogastric tube re-insertion. High-output stoma was defined as > 1200 mL/day after return to regular diet. Anastomotic leak was considered for any septic event clinically relevant related to an anastomosis and confirmed with a CT-scan study (perianastomotic fluid collection, abnormal pneumoperitoneum, or contrast extravasation). Secondary outcomes for this study included disease progression between the two CRS, disease-free survival (DFS), and overall survival (OS).

### Statistical analysis

Standard descriptive statistics were used to report patients’ characteristics and perioperative events. Quantitative measures were described as mean (standard deviation) or median and range (minimum and maximum values) were reported. Qualitative values were reported as percentage. Software used for descriptive statistics was Excel (Microsoft, Redmond USA).

## Results

Between January 2012 and March 2020, 78 patients operated for PMP arising from the appendix were identified. Of those, 8 patients underwent the two-step approach and were included in the study. Baseline patient characteristics are presented in Table 1. A majority of patients presented with synchronous peritoneal metastases (PM) and significant visible disease on preoperative imaging. Clinical and pathological characteristics are presented in Table 2.

**Table 1:** Baseline characteristics

Variable	Overall
*N*	8
Age (years)	60.5 [40.0–69.0]
Gender	
Male	1 (12.5)
Female	7 (87.5)
BMI (kg/m2)	23.6 [19.8–34.0]
ASA class	
I	1 (12.5)
II	6 (75.0)
III	1 (12.5)

Values are presented as median [min-max range] or *n* (%). *BMI* body mass index, *ASA* American Society of Anesthesiology

**Table 2:** Clinical and pathological characteristics at initial presentation

Variable	*n*
Presentation	
Synchronous peritoneal metastases	6 (75.0)
Metachronous peritoneal metastases	2 (25.0)
CT Scan findings	
No CT	0 (0)
Mild disease	2 (25.0)
Significant disease	6 (75.0)
Preoperative laparoscopy	3 (37.5)
Pathology (PSOGI)	
LAMN	7 (87.5)
HAMN	1 (12.5)
Pathology (Ronnett)	
DPAM	4 (50.0)
PMCA-I	4 (50.0)
Neoadjuvant chemotherapy	1 (11.1)

Values are presented as *n* (%). *PSOGI* Peritoneal Surface Oncology Group International, *LAMN* low-grade appendiceal mucinous neoplasms, *HAMN* high-grade appendiceal mucinous neoplasms, *DPAM* diffuse peritoneal adenomucinosis, *PMCA-I* peritoneal mucinous carcinomatosis with intermediate features

Intraoperative outcomes are presented in Table 3. Procedures were performed by a team of two attending surgeons and two residents, with one surgeon present at both steps for each patient. The median PCI at initial laparotomy was 33 (29–39), while the median PCI at second laparotomy was 21 (15–39). The median time between the two procedures was 111 days. Complete CRS (CC-0 or CC-1) at the second procedure was achieved for 7 patients, while one patient was deemed unresectable at second laparotomy and therefore did not undergo CRS and HIPEC. Median duration of both procedures was 7 and 8 h, respectively, whereas estimated blood loss for both procedures was 800 and 1100 mL, respectively.

**Table 3:** Intraoperative outcomes

Variable	1st CRS	2nd CRS
Time to OR^a^ (days)	109 [22–216]	111 [90–212]
PCI	33 [29–39]	21 [15–39]
CRS performed	8 (100)	7 (87.5)
HIPEC performed	0 (0)	7 (87.5)
Resection		
Hollow viscera	2 [1–3]	1 [0–2]
Solid viscera	2 [0–3]	2 [0–3]
Anastomoses	1 [1–2]	0 [0–2]
Completeness of cytoreduction		
0	0 (0)	5 (55.6)
1	0 (0)	3 (33.3)
2	8 (100)	0 (0)
Not resectable	0 (0)	1 (11.1)
Duration of procedure (min)	420 [180–530]	480 [180–660]
Estimated blood loss (mL)	800 [300–4000]	1100 [500–2700]
*n* patient transfused	3 (37.5)	5 (62.5)

Values are presented as median [range] or *n* (%)

*CRS* cytoreductive surgery, *OR* operating room, *PCI* Peritoneal Cancer Index, *HIPEC* hyperthermic intraperitoneal chemotherapy

^a^Time from diagnosis to 1st CRS, and time from 1st to 2nd CRS, respectively

Postoperative outcomes are presented in Table 4. There was no postoperative mortality in this series. After the first procedure, two patients were admitted to the intensive care unit (ICU) for a maximum of 2 days, and the median time to resume diet was 3 days (1–11 days). No patient experienced a major complication, while 50% had minor complications. The median length of stay was 16 days (10–17 days). After the second procedure, 6 patients were admitted to the ICU for a median of 2 days (0–6 days). The median time to resume diet was 9 days (1–14 days). The rate of adverse events was 62.5% for minor complications and 25% for major complications, including one patient who underwent a re-operation for intra-abdominal abscess.

**Table 4:** Post-operative outcomes

Variable	1st CRS	2nd CRS
ICU (days)	0 [0–2]	2 [0–6]
Time to resume diet (days)	3 [1–11]	9 [1–14]
TPN (days)	5 [0–14]	12.5 [0–27]
Complications		
Minor (CD I-II)	4 (50.0)	5 (62.5)
Major (CD III-V)	0 (0)	2 (25.0)
Prolonged ileus	1 (12.5)	2 (25.0)
Pulmonary morbidity	0 (0)	2 (25.0)
Delayed gastric emptying	0 (0)	2 (25.0)
Urinary tract infection	2 (25.0)	2 (25.0)
Intraabdominal abscess	0 (0)	1 (12.5)
Deep vein thrombosis	1 (12.5)	2 (25.0)
Reoperation	0 (0)	1 (12.5)
High output ileostomy	0 (0)	1 (12.5)
Gastric leak	1 (12.5)	0 (0)
SIADH	1 (12.5)	0 (0)
Length of stay (days)	16 [10–17]	19 [10–41]
Operative mortality	0 (0)	0 (0)
Follow-up duration (months)	-	53.8 [3–73]

Values are presented as median [range] or *n* (%). *ICU* intensive care unit, *TPN* total parenteral nutrition, *CD* Clavien-Dindo Classification; *SIADH* syndrome of inappropriate antidiuretic hormone secretion

The median follow-up was 53.8 months (3–73 months), starting from the second procedure. The details for each patient are presented in Table 5. Two patients had a recurrence. The first one had a DFS of 16.6 months and an OS of 66 months. The second patient had a DFS of 9.3 months, but was still alive at 63 months. The patient who was deemed unresectable at second surgery survived 39 months with palliative care.

**Table 5:** Patients’ detailed characteristics and follow-up

Patients	Histology	PCI (1st CRS)	PCI (2nd CRS)	Time between procedures (days)	Time to relapse (months)	Time to death (months)	Follow-up (months)	Current status
1	HAMN	31	23	162	16.6	66	66	Dead
2	LAMN	34	16	120	-	-	73	Alive
3	LAMN	33	22	90	9.3	-	63	Alive with recurrence
4	LAMN	39	15	91	-	-	63	Alive
5	LAMN	39	39	119	NA	-	39	Dead
6	LAMN	39	21	98	-	-	45	Alive
7	LAMN	29	15	115	-	-	23	Alive
8	LAMN	30	25	107	-	-	3	Alive

*PCI* Peritoneal Cancer Index, *CRS* cytoreductive surgery, *HAMN* high-grade appendiceal mucinous neoplasms, *LAMN* low-grade appendiceal mucinous neoplasms, *NA* not applicable

## Discussion

This study reports the outcome of 8 PMP patients with very high PCI who were approached with a two-step surgical strategy in an attempt to reduce the postoperative morbidity and mortality associated with CRS for such an extensive disease. While one patient was deemed unresectable at the second surgery, complete CRS with HIPEC was feasible for 7 patients, with good oncological outcomes, reasonable morbidity, and no mortality.

Patient selection can be challenging in PMP cases with very high PCI. Predicting resectability with preoperative imaging remains an issue [21] and the need to resect multiple organs or digestive segments [22] is particularly difficult to anticipate. Furthermore, extensive disease [23, 24], prolonged operative time, and postoperative complications are all factors associated with long-term impairment of quality of life [10], which has to be considered when performing CRS and HIPEC for such patients. Since the only therapeutic alternatives are debulking and systemic chemotherapy, which are both palliative options [25], CRS and HIPEC in this population may be considered in a curative intent, but only if a complete CRS is achievable with acceptable postoperative morbidity and impact on quality of life.

Benhaim et al. [17] recently published a series of 68 PMP patients with extensive disease that underwent complete CRS with HIPEC in a single procedure, with a median PCI of 37 (28–39). The mean operative time was 713 min (± 119 min), mean estimated blood loss was 1826 mL and mean hospital stay was 37 days (± 24 days). Finally, severe complications and mortality were reported in 46% and 8% of patients, respectively. In the present study, the two-step approach resulted in a median PCI reduction of 12 (33 to 21) between the two procedures, with a combined mean operative time of 856 min (min-max range 600–1080 min), estimated blood loss of 2650 mL (min-max range 1100–5200 mL) and hospital stay of 37 days (min-max range 26–53 days). However, severe complications were reported in only 25% of patients with no mortality and only at the second surgery. This could be explained by the longer length of the procedure and the higher estimated blood loss after iterative laparotomy. Morbidity and mortality rates of the two-step approach are consistent with morbidity rates (2.7–33%) and mortality rates (0–2.7%) reported in the literature [26] for all PMP patients, regardless of the PCI. We could hypothesize that in a two-step approach, operative outcomes are cumulative, but morbidity and mortality are not, with each procedure having its own risks and associated complications. To ascertain this hypothesis, comparison of a two-step approach with other approaches should be performed.

Only one other example of two-step approach for peritoneal surface malignancy has been reported in the literature. Leinwand et al. [27] reported a series of 204 cases of malignant peritoneal mesothelioma treated with a similar strategy. The first step consisted of an omentectomy and gross debulking, followed by the insertion of an intraperitoneal catheter for chemotherapy infusion in the postoperative course, with a planned second look. The second surgery was performed in 63% of the patients. HIPEC was delivered in 61% after the first CRS and 89% after the second CRS. Major complications were reported in 13% of patients after the first surgery and 10% after the second surgery. PCI was not reported. The median time between the two procedures was 5.4 months (interquartile range (IQR) 4.8–6.0 months). Although our approach focused on a different peritoneal surface malignancy (PMP) and disease severity assessment was different, reported complication rates were comparable.

In our series, two patients had a recurrence. One patient presented with peritoneal recurrence at 9.3 months after a complete two-step surgery and underwent a third intervention consisting of CRS and mitomycin C HIPEC. This patient is still alive after 63 months of follow-up. Another patient presented with peritoneal recurrence at 16.6 months but was deemed unresectable at exploratory laparotomy. Despite receiving palliative chemotherapy, the patient died at 66 months. For that patient, the initial histology of peritoneal metastases was low-grade PMP, but revision of pathology slides later classified the disease as high-grade PMP. The aggressiveness of the disease was therefore higher than initially expected and might have been the cause of the unfavorable outcome. This highlights the fact that this two-step approach should be reserved for low-grade disease, as high-grade disease presents a higher risk of early recurrence [28]. In total, at follow-up, 5 patients (62.5%) were alive without disease recurrence, which can be considered comparable to the 5-year disease-free survival reported by Benhaim et al. [17] for extensive PMP patients operated on with a one-step approach (45%).

Optimal timing for the second procedure has yet to be determined. The hypothesis is that the surgical risk of an early reoperation is outweighed by the risk of progression, as PMP is an indolent disease. The patient needs to recover completely from the first surgery. Complete resorption of the postoperative intraabdominal inflammatory response is necessary in order to proceed to second CRS. In this series, time between the two procedures ranged from 90 to 212 days and no patient was deemed unresectable because of an unresolved inflammatory response. However, one patient PMCA-I disease (PSOGI low-grade PMP) presented an early invasive pelvic recurrence after the initial surgery, with a time span of 4 months between the two procedures. Because of the early recurrence and overall extent of the disease, CRS and HIPEC were not performed. Palliative chemotherapy was started and the patient eventually died 39 months later. Having said that, it is noteworthy that the patient was symptomatic (bloating and abdominal discomfort) before his first surgery and felt much better after, without severe morbidity. In the recent guidelines published by PSOGI/EURACAN (European Network for Rare Adults Solid Cancer) [26], tumor debulking was an accepted strategy for selected patients with unresectable low grade PMP, as it may improve quality of life with acceptable morbidity and mortality. Even though the patients undergoing the two-step approach are operated with a curative intent, the first step certainly reduces the bulkiness of the disease and may improve patients’ condition in a similar way than palliative tumor debulking.

This study is not without its limitations. This is a retrospective study and only a small number of patients could be included, as extensive PMP is a rare presentation of a rare disease. No group-control comparison was possible within our institution, since all PMP patients with very high PCI are approached with a two-step strategy. Therefore, safety and oncologic outcomes could not be compared to a one-step CRS or palliative approach. Future studies aiming to compare the outcomes of PMP patients with very high PCI after pairing them with patients from large international cohorts could be helpful.

## Conclusion

A two-step surgical management for low-grade PMP patients with very high PCI seems to be safe and feasible, with reasonable morbidity and no compromise on oncological outcomes. It may be considered for PMP patients with extensive disease, for which prolonged operative time and important surgical morbidity is expected.

## Data Availability

Data are available from the authors upon request to Dr. Lucas Sidéris.
